# Strengthening health system leadership for better governance: what does it take?

**DOI:** 10.1093/heapol/czy052

**Published:** 2018-07-08

**Authors:** Lucy Gilson, Irene Akua Agyepong

**Affiliations:** 1 Health Policy and Systems Division, School of Public Health and Family Medicine, University of Cape Town, Anzio Road, Observatory, South Africa; 2 Department of Global Health and Development, London School of Hygiene and Tropical Medicine, Keppel Street, London, UK; 3 Division of Research and Development, Dodowa Health Research Centre, Box DD1, Dodowa, Ghana; 4 Department of Health Policy, Planning and Management, School of Public Health, University of Ghana, Legon, Accra, Ghana

**Keywords:** Leadership, governance, health systems, leadership development, Africa

## Abstract

This editorial provides an overview of the six papers included in this special supplement on health leadership in Africa. Together the papers provide evidence of leadership in public hospital settings and of initiatives to strengthen leadership development. On the one hand, they demonstrate both that current leadership practices often impact negatively on staff motivation and patient care, and that contextual factors underpin poor leadership. On the other hand, they provide some evidence of the positive potential of new forms of participatory leadership, together with ideas about what forms of leadership development intervention can nurture new forms of leadership. Finally, the papers prompt reflection on the research needed to support the implementation of such interventions.

**Key Messages**

- Leadership practices influence staff motivation and team work, with consequences for patient care
- Current health system contexts commonly encourage negative leadership practices
- Strengthening health leadership is a system-wide reform that requires intervention at individual, team and system levels
- Future leadership research must acknowledge and engage with context in understanding how to strengthen health systems

## Introduction

This special supplement adds to the still limited knowledge base about health leadership in low- and middle-income countries (LMICs) and more specifically, in African settings. The six papers presented here are all written by predominantly African author teams. Three papers consider experiences in public hospitals in different countries, and three consider different experiences of health leadership development. Together they add to the smattering of existing empirical and experiential work about African health leadership (Kebede *et al*. 2009; Curry *et al*. 2012; Doherty *et al*. 2013; Chigudu *et al*. 2018; Gilson *et al*. 2014; Kwamie *et al*. 2014; Omaswa and Crisp, 2014; Bradley *et al*. 2015; Choonara *et al*. 2017; Mutale *et al*. 2017).

## What is the importance of leadership, and related research, for health system development?

Building on long-standing concern for district health management (Cassels and Janovsky 1991; Conn *et al.* 1996), the 2000 WHO Report on Health Systems (WHO 2000) brought new attention to the importance of ‘stewardship’ as a critical element of every health system. Later relabelled ‘leadership and governance’ (WHO 2007, 2008), this element has increasingly been recognized as a critical lever for health system development (de Savigny and Adams 2009; Frenk 2010). Indeed, longitudinal analyses of health system change in specific settings, such as Thailand (Tangcharoensathien 2018), as well as cross-country analyses (Balabanova *et al.* 2013; Samuels *et al.* 2017), have demonstrated this leverage role. The particular importance of leadership, specifically, was also affirmed by the Alliance for Health Policy and Systems Research (AHPSR) 2016 Flagship Report ‘Open Mindsets: Participatory Leadership for Health’ (AHPSR 2016).

However, current (2018) debates about governance do not always give attention to the role of leadership within governance. Instead they often focus on, e.g. the institutions and rules influencing governance, or on governance reforms such as decentralization or local accountability (Fryatt *et al.* 2017; Pyone *et al.* 2017).

A key challenge to thinking about leadership may be that relevant empirical evidence from LMICs remains scanty. The AHPSR thus called for ‘multidisciplinary research on the nature, quality and contributions of leadership in health systems’ (AHPSR 2016, p. 9; see also Reich *et al.* 2016).

## What do the papers presented here consider?

All the papers in the special supplement acknowledge that leadership and management can be seen as distinct, but related, areas of competence, and that health managers must always be ‘managers that lead’ (Galer *et al.* 2005). Where managing entails a focus on coordinating resources and implementing activities to produce reliable performance, leading is about enabling those within and outside the system to face challenges and achieve results under complex conditions (Scholtes 1998).

Three papers presenting evidence on the role of, and influences over, leadership and management within public hospitals are drawn from three different countries. A South African analysis of two rural case study hospitals (Mathole *et al.*, 2018) shows how leadership style and practices can make a difference in terms of hospital performance. In contrast, the Ghanaian and Kenyan studies presented here illuminate quite negative pictures of public hospital leadership and management. Comparing two urban district hospitals in Ghana, one with specialist services and one without, Aberese-Ako *et al.* (2018) explore what strategies managers working at all levels of the hospitals used to cope with the contextual constraints they faced. In Kenya, meanwhile, Nzinga *et al.* (2018) working in two county (district) hospitals, reveal the individualized decision-making and often intimidatory leadership practices of mid-level managers, the clinical heads and nurses in charge of inpatient wards.

The other three papers in this edition offer insights into health leadership development needs and strategies:

- Agyepong *et al.* (2018) report on needs assessment and other preparatory work undertaken in Ghana, South Africa and Uganda, towards the development of a pan-African professional Doctorate in Public Health (DrPH), as a professional, interdisciplinary terminal degree focussed on strategic health leadership;
- Doherty *et al* (2018) present an evaluation of a well-established South African public sector health leadership development programme offering a combination of residential training plus workplace-based learning activities, at the level of a post-graduate Diploma in Health Management;
- Cleary *et al.* (2018) detail the experience of developing tailor-made leadership development (LD) processes to support relational (Cummings *et al.* 2010) and distributed (Gronn 2002) leadership within a sub-district setting in Cape Town, South Africa.

## What lessons can be drawn from this set of papers?

Taken together these papers illuminate, first, the nature of existing public sector leadership practices in various African health system settings—showing that individual leaders often exercise power in quite authoritarian and hierarchical ways. They also clearly demonstrate that, across countries, the existing public sector context provides barriers to exercising the sort of participatory leadership called for by the Alliance for Health Policy and Systems Research (2016). Among these barriers are not only resource shortages, but also features of organizational culture such as centralized decision-making, individualized decision-making and medical professionals’ dominance.

However, rather than concluding that participatory and distributed leadership is an idealistic goal for public health systems as currently structured, these experiences simply demonstrate that the status quo must be disrupted.

As the papers show, ‘current’ leadership practices have ‘negative’ consequences for staff motivation, professional practice and patient care. Yet the papers also provide evidence of the ‘positive potential of new forms of participatory leadership’—in terms of encouraging teamwork and relationships, tackling problems collectively, spreading motivation and positive staff attitude. Senior managers consulted about the possible pan-African DrPH programme confirmed, moreover, that such leadership is what is needed in every health system (Agyepong *et al.* 2018). The particular importance of participatory leadership for health systems lies in their complexity—they are comprised of multiple sets of people and organizations working within a dynamic environment of changing health needs, medical and technological advances and resource conditions. As in any complex system, leadership is necessary not only to guide and enable the different parts of the system to work towards common goals (Chunharas and Davies 2016), but also to enable the emergence of learning, creative and adaptive capacity (Uhl-Bien *et al.* 2007); and such leadership is a collective product of leaders and followers co-constructing shared meaning and action towards common objectives (Bolden 2011). Thus,

*‘Strengthening leadership in health requires a focus on ensuring an eco-system that enables participation from diverse actors, nurtures debates and provides an opportunity for all actors to assert their leadership potential, as the need arises, to the benefit of improved health-system performance’* (AHPSR 2016, p. 6).

It is, then, essential to think differently about how to ‘do leadership development’, as current efforts are clearly not effective.

In this regard, these papers suggest that it is not enough to train individuals; instead it is necessary to engage workplace teams in leadership development programmes—especially given the inter-disciplinary requirements of health care and the need for inter-sectoral actions to promote health. It is also not enough to train leaders away from their workplaces; instead experiential skills and tacit knowledge must be developed within workplace teams. Finally, it is not enough to train people, it is necessary also to develop an organizational context that sustains new leadership practices. This requires both a critical mass of people with new leadership skills whose micro-practices of governance (Gilson *et al.* 2017) work to change the context from within, and new structures of governance that spread decision-making power and encourage multiple forms of accountability. In other words, leadership and governance are intertwined. And strengthening leadership in complex health systems is a system-wide reform, requiring collaboration between current health system leaders, educators and other groups (Cassels and Janovksy 1991).

Finally, leadership development can be supported by research. The complex interactions of leadership, context and system change illustrated in the papers presented here point to the particular importance of flexible and qualitative research (Gilson 2012). All three hospital studies drew, for example, on at least some ethnographic component to develop rich understanding of the experiences in their settings. Quasi-experimental studies simply cannot allow for the complex phenomena entailed in leadership and leadership development. Focussed on assessing whether or not an intervention works and can be replicated across settings, such studies simply address the wrong question. As a system-wide reform, the key focus of inquiry around health leadership development must be to understand how programme design enables system change in a particular context. For such research, critical realist evaluation has clear relevance (e.g. Kwamie *et al.* 2014; Prashanth *et al.* 2014). Cleary *et al.* (2018) also demonstrate the value of action learning processes that not only support co-design of interventions, but also generate insights about the experience and provide mechanisms for feeding these insights back into programme development (Lehmann and Gilson, 2015). Recognizing the complexity of health systems and of bringing change within them demands both context-specific health leadership development interventions and research that acknowledges and engages with that context.
